# Measuring and improving the quality of mental health care: bringing patient-reported outcome et experience measures (PROMs et PREMs) in psychiatric settings

**DOI:** 10.1192/j.eurpsy.2023.688

**Published:** 2023-07-19

**Authors:** E. Scanferla

**Affiliations:** CMME, GHU Paris Psychiatrie et neurosciences, PARIS, France

## Abstract

**Introduction:**

Patient-reported outcome and experience measures (PROMs and PREMs) are increasingly acknowledged as critical to enhancing patient-centred, value-based care. However, research is lacking on the function and relevance of these instruments in acute psychiatric care.

**Objectives:**

The main objective of this study was to evaluate the domain of subjective well-being as a relevant indicator of the quality of hospital care, distinct from measures of symptom improvement and satisfaction with care reported by patients, assuming they only incompletely reflect inpatients’ unmet needs and expectations. [YA5] [SE6]

We hypothesised that the patients’ measures of subjective well-being (generic PROMs) at discharge are only partially related to the satisfaction with the experience of care (PREMs), that in turn differs from the clinician’s experience of the provided care, and symptom improvement (disease-specific PROMs).

**Methods:**

Two hundred and forty-eight inpatients of a psychiatric university hospital were included in the study between January and June 2021. Subjective well-being was assessed using standardised generic PROMs on well-being, symptom improvement was assessed using standardised disease-specific PROMs, and experience of care with PREMs. PROMs were completed at admission and discharge, PREMs were completed at discharge. Clinicians rated their experience of provided treatment using adapted PREMs items.

**Results:**

Change in subjective well-being (PROMs) at discharge was significantly (p<.001), but moderately (r²=28.5%), correlated to improvement in symptom outcomes, and weakly correlated to the experience of care (PREMs) (r²=11.0%), the latter being weakly explained by symptom changes (r²=6.9%. Patients and clinicians assessed differently the experience of care.

**Conclusions:**

Findings confirmed our hypothesis showing that across mental disorders improvement in subjective well-being was weakly correlated to the experience of care and moderately, negatively, correlated to symptom outcomes. Improvement in symptoms was found to be the strongest predictor of increase in subjective well-being at discharge, but it explained only a moderate part of its variance.

In conclusion, this study shows that PROMs and PREMs have potential as key indicators of high quality care across mental health services, and supports the case for measuring subjective well-being as a relevant indicator in its own right, particularly in psychiatric hospital treatments. Patient-reported measures enable the implementation of patient-centred therapeutic management and the delivery of services focused on achieving outcomes that matter to patients across the full pathway of care.

**Disclosure of Interest:**

None Declared

